# Improving outcomes for hospitalised First Nations peoples through greater cultural safety and better communication: the Communicate Study Partnership study protocol

**DOI:** 10.1186/s13012-023-01276-1

**Published:** 2023-06-22

**Authors:** Anna P. Ralph, Stuart Yiwarr McGrath, Emily Armstrong, Rarrtjiwuy Melanie Herdman, Leah Ginnivan, Anne Lowell, Bilawara Lee, Gillian Gorham, Sean Taylor, Marita Hefler, Vicki Kerrigan

**Affiliations:** 1 Menzies School of Health Research, Charles Darwin University, Darwin, NT Australia; 2 Department of Medicine, Royal Darwin Hospital, Darwin, NT Australia; 3 Northern Institute, Charles Darwin University, Darwin, Australia; 4 Djalkiri Foundation, Nhulunbuy, Australia; 5 Miwatj Health Corporation, Nhulunbuy, Australia; 6 Department of Women’s and Children’s Health, Royal Darwin Hospital, Darwin, NT Australia; 7 Indigenous Leadership, Charles Darwin University, Darwin, Australia; 8 Northern Territory Government Department of Health, Darwin, Australia

## Abstract

**Background:**

The Communicate Study is a partnership project which aims to transform the culture of healthcare systems to achieve excellence in culturally safe care for First Nations people. It responds to the ongoing impact of colonisation which results in First Nations peoples experiencing adverse outcomes of hospitalisation in Australia’s Northern Territory. In this setting, the majority of healthcare users are First Nations peoples, but the majority of healthcare providers are not. Our hypotheses are that strategies to ensure cultural safety can be effectively taught, systems can become culturally safe and that the provision of culturally safe healthcare in first languages will improve experiences and outcomes of hospitalisation.

**Methods:**

We will implement a multicomponent intervention at three hospitals over 4 years. The main intervention components are as follows: cultural safety training called ‘Ask the Specialist Plus’ which incorporates a locally developed, purpose-built podcast, developing a community of practice in cultural safety and improving access to and uptake of Aboriginal language interpreters. Intervention components are informed by the ‘behaviour change wheel’ and address a supply–demand model for interpreters. The philosophical underpinnings are critical race theory, Freirean pedagogy and cultural safety. There are co-primary qualitative and quantitative outcome measures: cultural safety, as experienced by First Nations peoples at participating hospitals, and proportion of admitted First Nations patients who self-discharge. Qualitative measures of patient and provider experience, and patient-provider interactions, will be examined through interviews and observational data. Quantitative outcomes (documentation of language, uptake of interpreters (booked and completed), proportion of admissions ending in self-discharge, unplanned readmission, hospital length of stay, costs and cost benefits of interpreter use) will be measured using time-series analysis. Continuous quality improvement will use data in a participatory way to motivate change. Programme evaluation will assess Reach, Effectiveness, Adoption, Implementation and Maintenance (‘RE-AIM’).

**Discussion:**

The intervention components are innovative, sustainable and have been successfully piloted. Refinement and scale-up through this project have the potential to transform First Nations patients’ experiences of care and health outcomes.

**Trial registration:**

Registered with ClinicalTrials.gov Protocol Record 2008644

**Supplementary Information:**

The online version contains supplementary material available at 10.1186/s13012-023-01276-1.

Contributions to the literature

- This paper describes the theories and methods for the Communicate Study Partnership in Northern Australia. This study aims to transform healthcare systems to achieve excellence in culturally safe care for First Nations peoples.
- Existing evidence and relevant implementation science approaches are presented to explain why we anticipate the study interventions will achieve the desired outcomes.
- The three main intervention components comprise the following: (1) cultural safety training entitled ‘Ask the Specialist Plus’ incorporating an innovative podcast to inspire critical reflection, (2) fostering a community of practice in cultural safety among healthcare providers, and (3) improving access to and uptake of Aboriginal language interpreters.

## Introduction

In Australia’s NT (NT), the repercussions of colonisation on First Nations people are evident in healthcare [1, 2]. Healthcare providers working in the tertiary sector can feel removed from the responsibility of ‘closing the gap’ in First Nations health disadvantage [1], seeing this as a responsibility of government or primary care. But those working at all levels of the health system have the power to contribute effectively to closing the gap. The Communicate Study Partnership has identified problem points within tertiary healthcare settings affecting First Nations peoples’ experiences of care and outcomes and has devised practical solutions to achieve greater cultural safety in service delivery. Our hypotheses are that cultural safety can be effectively taught, systems with entrenched ways of operating can change to become culturally safe and that more culturally safe care will be associated with better health outcomes.

The rationale for this project includes current low uptake of interpreters despite First Nations languages being spoken [3], racism in healthcare [4, 5], high rates of ‘take own leave’ from hospitals (10–12% of admissions [6], around 11 times the rate for non-Aboriginal people [7]) and high mortality [8]. Meanwhile, healthcare providers recognise they lack skills in delivering culturally safe care [9] and have requested training to address their limitations [10].

Australian governments have committed to addressing racism in healthcare by endorsing culturally safe care [11, 12]. However, a policy-practice gap exists. Our research focuses on implementing cultural safety to support healthcare professionals to develop ‘critical consciousness’ [13] and to provide care which is free from stereotypical thinking [14]. We estimate that First Nations language interpreters should be used for around 50% of First Nations patients in the NT Top End, but only around 18% are currently getting access [15]. Use of professional interpreters improves patient outcomes [16, 17]. Interpreters are underutilised in the NT due to supply and demand factors [3]. Low demand for interpreters can be attributed to lack of knowledge about the diversity and prevalence of languages spoken in the NT and time constraints in acute care work environments [3]. Hospitals can be alienating places for interpreters, reducing their motivation to accept interpreting jobs at the hospital [5, 9]. Healthcare providers may lack skills in how to work effectively with interpreters [3]. Therefore, interpreters and other First Nations staff (liaison officers, health practitioners) require culturally safe workplaces, mentoring and career pathways, and healthcare providers require training in working with First Nations staff.

The Communicate Study, developed in response to these identified policy-practice gaps, is a partnership between Menzies School of Health Research, the NT Government Department of Health, the NT Aboriginal Interpreter Service and the National Accreditation Agency for Translators and Interpreters. Aims are to transform healthcare systems to achieve excellence in providing culturally safe care for First Nations people, strengthen the tools and strategies underpinning culturally safe practice and measure outcomes using comprehensive qualitative and quantitative measures.

## Methods

This is a multicomponent intervention with three aims (Table 1) guided by implementation science approaches (Table 2). Major components of the intervention, described using the TIDieR Checklist (Template for Intervention Description and Replication) [18] (Supplementary Table), are as follows: cultural safety training, a community of practice of cultural safety champions, employment of and education for interpreters and advocacy strategies for cultural change within health systems to provide more fit-for-purpose care for the majority First Nations patients.

**Table 1:** The Communicate Study Partnership aims

**Aim 1: Transform the culture of healthcare systems to achieve excellence in providing culturally safe care for First Nations peoples** a.Develop, implement and evaluate anti-racism training using Ask the Specialist Plus. This comprises moderated discussion and reflection on ‘Ask the Specialist’ podcast episodes held during in-service and clinical teaching timeslots for healthcare providers b.Create a community of practice of culturally safe clinicians supported by a social media chat group and seminars provided by invited experts
**Aim 2: Strengthen the tools and strategies required underpinning culturally safe practice** a.Improve **demand** for Aboriginal interpreters and Aboriginal health practitioners through improved knowledge of language diversity and cultural safety and recognition of patient needs b.Improve **supply** of interpreters and Aboriginal health practitioners willing to work in the hospital environment by creating a culturally safe workplace and supporting career pathways c.Tailor **effectiveness** strategies to participating sites such as the following: •Positioning interpreters at points of need and embedding them in medical and surgical teams •Optimising workflow to facilitate efficiency and availability across hospital departments
**Aim 3: Evaluate outcomes using comprehensive qualitative and quantitative measures** a.Qualitative enquiry to assess cultural safety from patient perspectives and understand experiences of Aboriginal and non-Aboriginal healthcare providers and interpreters b.Quantitative outcomes including the following: •Performance across key indicators: Changes in documentation of language, interpreter bookings made, interpreter bookings completed and % Aboriginal patients in need getting access to an interpreter •Impact of intervention: Proportion of admissions with and without interpreters ending in self-discharge, unplanned re-admissions and changes in hospital length of stay •Economic analysis of the costs and cost benefits of interpreter use to decrease self-discharge and re-admission rates

**Table 2:** Methodological approaches used in the Communicate Study Partnership

Category	Philosophical theories	Implementation theory	Determinant framework	Process models	Evaluation
Specific approaches	•Cultural safety •Critical race theory •Freirean pedagogy •Whiteness studies •Decolonising theory	•Behaviour change wheel	•Supply–demand-efficiency-effectiveness cycle	•Participatory action research •Continuous quality improvement	•RE-AIM •Kirkpatrick model of learning
Description	Together, these provide philosophical and ethical guidance	Describes the breadth of interventions needed for successful behaviour change among healthcare providers	Describes the elements required to improve uptake of interpreters, where supply refers to interpreter availability, demand and effectiveness refer to healthcare provider behaviours and capabilities and efficiency relates to system factors	These methods guide translation of research into practice, using a participatory approach: proactive engagement of relevant front line and executive staff from health and interpreter services with data to motivate improvement in cultural safety performance indicators	Provides a structured way to critique the programme to summarise successes, failures and learnings
Rationale for choice of specific approach	Appropriate to the subject matter of First Nations healthcare in the predominant White Australian health system context	This project focuses on changing the behaviours of healthcare providers and changing institutional culture	These are the determinants we hypothesise will influence success in delivering culturally safe care and other implementation outcomes	This approach will support long-term sustainability	These are both validated, pragmatic ways to report overall programme outcomes and training outcomes respectively

**Fig. 1:**
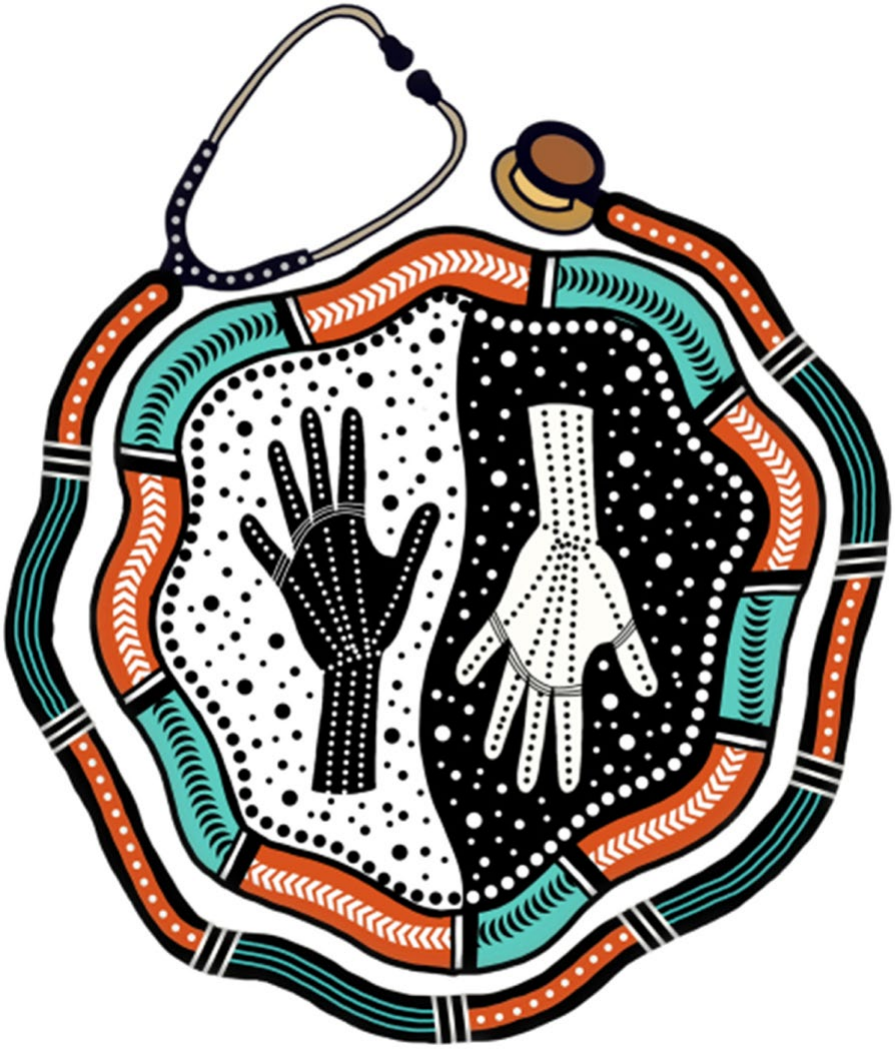
Artwork created by Jason Lee to illustrate the Communicate Study

The artwork to illustrate the study was created by Larrakia artist Jason Lee (Fig. 1). Lee states the following: “The two hands represent the relationship between doctor and patient. The inner ring represents Menzies and the community and male and female patients.”

### Design

**Fig. 2:**
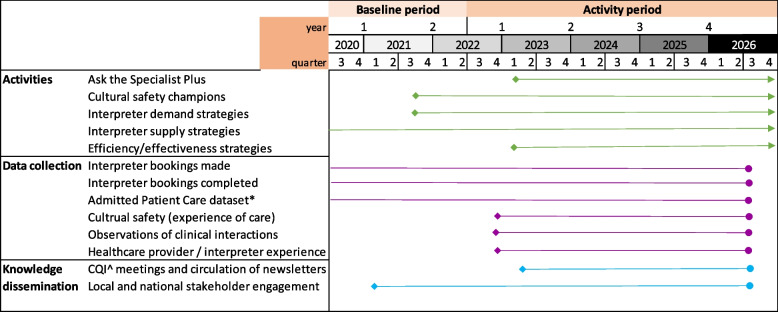
Study timeline *includes length of stay, self discharge, readmission; ^CQI: continuous quality improvement; Coloured lines: arrow indicates sustainable activites beyond project end; circle indicates completion of activities

A multicomponent intervention will be implemented over 4 years. Impact will be evaluated longitudinally using data collected during baseline periods prior to activity implementation and study implementation periods. The baseline period is July 1, 2020, to June 30, 2022 and activity period July 1, 2022, to June 30, 2026, for the purposes of quantitative data (Fig. 2). Additionally, individual before-and-after time frames for qualitative data collection will allow comparison of impacts of sequential training sessions run during 2023–2026.

The impact of the activities will be assessed using time-series analysis of quantitative outcomes and qualitative measures of patient-provider interactions and experience (Table 1 and details below). A continuous quality improvement (CQI) approach [19, 20] will ensure data are used pro-actively, through participatory methods that engage and motivate relevant front-line and executive staff from health and interpreter services. Outcome measures include cultural safety, as experienced by First Nations peoples treated at participating hospitals, and proportion of admitted First Nations patients who self-discharge (Table 3).

**Table 3:** Outcome measures, data sources and analytical approach

	**Outcome measure**	**What does this capture?**	**Data source**	**Analysis**
Quantitative	Documentation of language	Health system quality, safety, efficiency	Electronic medical records	Time-series analysis
	Documentation of interpreter need in patient records			
	Interpreter bookings made	Healthcare provider behaviour	Linked admitted patient care (APC) and Aboriginal Interpreter Service (AIS) dataset	
	Interpreter bookings completed	System efficiency and interpreter willingness		
	% Aboriginal patients in need^a^ getting access to an interpreter	Combination of the above		
	Self-discharge	Patient health outcome		
	Unplanned readmission	Health system cost Patient health outcome		
	Hospital length of stay	Health system cost		
Survey data	Surveys after participating in each session of ‘Ask the Specialist Plus’	Value of training and appropriateness of podcast format	Surveys to assess reaction to training, skills obtained, application, benefit	Descriptive summary data
	Count of NT Health interpreters and AHPs employed and retained; turnover in role during the 5 years of the study; languages represented	Quality of support for Aboriginal staff; institutional cultural safety	NT Health employment records	Annual report of descriptive summary data
	NAATI certification status of interpreters — % having attained levels 1, 2, 3 or full certification	Effectiveness of interpreter training	AIS records	Annual report of descriptive summary data
Qualitative	Cultural safety as experienced by First Nations Australian people managed at participating health services	Effectiveness of activities as perceived by practitioners and recipients of care	Interviews and observational data	Narrative analysis
	Healthcare provider experience			
	Interpreter/Aboriginal Health Practitioner experience			

^a^ *Proportion of Aboriginal patients in need of an interpreter*. Community consultation indicates that most people who speak an Aboriginal language as their first language would benefit from an interpreter in healthcare interactions; some advise an opt-out approach, offering an interpreter for anyone with an Aboriginal first language (podcast episode 2 [21]). We conservatively estimate 50% of Aboriginal patients would benefit from an interpreter. This project will provide better estimates of the true proportion

Overarching program evaluation will use the RE-AIM model (Reach, Effectiveness, Adoption, Implementation, Maintenance — Table 4) [22].

**Table 4:** Application of the RE-AIM framework to this project

RE-AIM dimension and operational definition	Plan	Indicators
How will I and the intervention reach the targeted population? *Reach is the absolute number or proportion of individuals who are willing to participate in a given initiative*	•The project team has First Nations leadership with cultural advice and community engagement fostered by First Nations members of the investigator team and project staff •Relevant stakeholders at participating service organisations are all involved as project partners •Members of the project team work within the services directly with the target populations •Healthcare providers are directly reached through cultural safety training sessions, being part of a social media chat group and attending evening seminars on cultural safety, providing direct reach •We will build on the already established connections and relationships we have	•Number of health providers who participate in training, seminars, observations and interviews and who become cultural safety champions •Number of people who participate in continuous quality improvement meetings •Number of interpreters employed, and staying in employment, and who participate in training and interviews •Number of patients who participate in sharing stories about their experiences of healthcare
How do I know my intervention is effective? *Effectiveness is the impact of an intervention on outcomes, including potential negative effects, quality of life and economic outcomes*	•Qualitative and quantitative outcomes will capture effectiveness of study activities in improving the following: oCultural safety, as determined by patients oPatient hospitalisation outcomes oHealthcare provider capabilities and attitude and behaviour change oWorkplace experience for interpreters and healthcare providers	•Number of patients with the following -Documentation of language -Documentation of interpreter need •Interpreter bookings -Made -Completed •% Aboriginal patients in need getting access to an interpreter •Hospitalisation outcome -Self-discharge -Unplanned readmission -Hospital length of stay -Cost •Post-training survey data (Kirkpatrick model) •Improved patient experience of care •Improved interpreter and healthcare provider workplace experience
How do I develop organisational support to deliver my intervention? *Adoption is the absolute number, proportion and representativeness of settings and intervention agents who are willing to initiate a programme*	•All partner organisations have given written commitment to improving cultural safety and promoting interpreter access •Study team members will present study findings and prepare policy and practice briefs to be tabled at organisational meetings and responded to (such as the NTG Aboriginal Health Committee) •Hospitals recognise that the project helps them reach required national standards (National Safety and Quality Health Service Standards) to achieve accreditation •The National Accreditation Authority for Translators and Interpreters has a goal of certifying more Aboriginal interpreters; this project will help achieve that aim	•Engagement in investigator meetings •Uptake of study newsletter (number of people who open the electronic newsletter) •Invitations to the study team to present at partner organisation seminars, grand rounds, committee meetings •Endorsement of study outputs and uptake of policy and practice briefs provided by the study
How do I ensure the intervention is delivered properly? *Implementation refers to the intervention agents’ fidelity to the various elements of intervention’s protocol*	•The project receives cultural guidance from inception through to implementation and analysis and dissemination from First Nations elders and leaders, who provide expert cultural and language advice and guidance •The project is run by an experienced team of study investigators, with a qualified project manager and research assistants •The project team is supported by research institutional structures to ensure appropriate operations (business manager, ethics committee and financial manager) •Activities, milestones and implementation issues will all be logged on an ongoing basis •Quarterly investigator meetings and continuous quality improvement meetings will provide mechanisms for keeping study implementation on track and adherent to the proposed methods •Weekly project team meetings will maintain momentum	•Activity log •Project milestone reporting to the funding agency •Annual reports to the ethics committee •The way in which intervention components may be tailored or modified during implementation will be documented in the implementation log
How do I incorporate the intervention, so it is delivered over the long term *Maintenance is the extent to which a programme or policy becomes institutionalised or part of the routine organisational practices and policies*	•The use of continuous quality improvement will embed practice locally at each participating site and sustainably •The implementation model will transition training activities from being run initially by the project team to being run by the participating services during the course of the study, especially as evidence of the value of the training mounts	•Transfer of facilitation of ‘Ask the Specialist Plus’ from the study team to the health services •Transfer of health training coordination and delivery for interpreters from the study team to the health service/Aboriginal interpreter service •Evidence of actions and advocacy by Cultural Safety Champions initiated independently of the project

### Ethical considerations

Regarding terminology, we use ‘First Nations’ which recognises the diversity of nations who hold unceded sovereignty over Australia. We use ‘Aboriginal’ as required per organisational naming conventions. We use the term ‘White’ for nonindigenous persons in keeping with definitions of White as a social category of people who, in societies with European-origin dominant cultures such as Australia, knowingly or unknowingly participate in a racialized society that positions them as superior [23, 24].

### Partnership model

Participating sites are hospitals in the ‘Top End’, NT, Australia: Royal Darwin Hospital (RDH), Katherine Hospital (KH), and Gove District Hospital (GDH). We use participatory action research (PAR) in which stakeholders, participants and researchers collaborate to develop and enact real-world solutions to complex problems within Top End hospitals [25]. PAR projects value the experiential knowledge of marginalised peoples; the model is increasingly used in health research with First Nations communities [26]. Communicate Study implementers report regularly to the Aboriginal Health Committee of the NT Government Department of Health, and the committees which guide the implementation of the Australian National Safety and Quality Health Service (NSQHS) Standards (‘partnering with consumers’, standard 2) and ‘communicating for safety’, standard 6).

### Philosophical frameworks and research theory

Decolonising philosophies informing project design include cultural safety [27, 28], critical race theory [29] and Freirean pedagogy [13]. Cultural safety is the provision of an environment and practices that are safe for peoples of all ethnicities. It is akin to antiracism, ‘about the analysis of power and not the customs and habits of anybody’ [27]. Cultural safety in healthcare is the responsibility of healthcare providers and institutions to learn and change, and the extent to which cultural safety is achieved is determined by patients. These philosophies are linked by the following: (1) a critical focus on colonisers, (2) foregrounding race and racism, (3) a commitment to social justice and participatory approaches, (4) the assertion that dialogue between seemingly disparate groups is paramount to creating societal change and (5) the understanding that individuals who develop critical consciousness are capable of identifying power dynamics and creating a more equitable society [13, 27, 29]. These philosophies are applicable to our research in healthcare because they focus on redressing the power imbalance between healthcare providers (in positions of power) and patients (whose status as patient compounds existing disempowerment and marginalisation), by encouraging development of new perspectives through dialogue (direct or indirect by way of podcasts or other forms of storytelling). A key concept is to share ‘counterstories’ (described in critical race theory as a strategy to dismantle racist thinking [30]) from First Nations peoples to challenge negative stereotypes. By engaging with counterstories, healthcare providers can reflect on the pervasive impacts of colonisation as the fundamental cause of ill health [31]. The interventions designed for healthcare providers (‘Ask the Specialist Plus’ training and the cultural safety champions group) encourage staff to question and reflect on medical culture, Whiteness, racism and colonisation [32].

The Freirean concept of ‘problem-posing education’ [13] has inspired our training interventions. Problem-posing education encourages learners to identify problems they face, in response to which teachers create relevant curricula to help solve those problems. It has been hypothesised that this approach can challenge the dominant paradigm by encouraging students to critically reflect rather than being deposited with information that reinforces the status quo [33]. This model was used to develop the Ask the Specialist podcast and the health education for hospital-based interpreters.

### Implementation theories and frameworks

Implementation theories, models and frameworks are summarised in Table 2. The ‘COM-B’ system (capability, opportunity, motivation) informs study activities, recognising these attributes are central to achieving behaviour change [34]. The Communicate Study activities have accordingly been devised to address capability, opportunity and motivation under the headings of supply, demand, efficiency and effectiveness. The ‘supply–demand’ cycle borrowed from the manufacturing sector serves as a relevant determinant framework of interpreter uptake [35]. *Supply* of Aboriginal interpreters requires recruitment of bilingual experts into employment as interpreters (*opportunity*), training, certification and mentoring (*capability*) and provision of rewarding career pathways (*motivation*). *Demand* is generated through more culturally safe health systems where cultural safety is prioritised and the benefits are visible and rewarded (*motivation*) and through better knowledge among healthcare providers that their clients speak diverse languages, about the names of those languages, and about how to access interpreters (*capability and opportunity*). *Efficiency* is achieved through simplified booking processes (*capability*); visibility of interpreters in areas of need such as wards, emergency, outpatient clinics and availability of enough interpreters across diverse Aboriginal languages (*opportunity*); and continuous quality improvement data feedback to motivate greater interpreter uptake (*motivation*). *Effectiveness* is achieved when healthcare providers are competent and trained in working with interpreters (*capability*), have time and space available for appropriate communication (*opportunity*), when healthcare providers and patients both see positive outcomes from interpreter use and when senior clinicians and executive leaders prioritise and value interpreter engagement (*motivation*).

A participatory approach will be used as noted, incorporating CQI that builds comprehensively on the plan-do-study-act cycle [36]. CQI will be achieved through quarterly meetings of key health service providers (clinical champions, working group members, heads of departments), investigators and project team members, to motivate practice change. Infographics and plots will be prepared by the project team to present quarterly data broken down by hospital and ward. This will include ‘cascades of care’ plots showing numbers of inpatients in relation to Aboriginal languages, interpreter bookings and booking completions. Length of stay, proportion of discharges ending in self-discharge, 30-day readmissions and key themes and quotations from qualitative data will also be shared in CQI sessions. A summary will be included in the quarterly study newsletter. These meetings will provide opportunities to identify and troubleshoot barriers and implement sustainable improvements through the partnership model with the relevant service providers.

### Activities to be implemented to address each aim

Activities are summarised in the Template for Intervention Description and Replication (TIDieR) checklist [18] (Supplementary Table) and described here in relation to each aim (Table 1).

### Achieve excellence in providing culturally safe care

Aim 1 is to transform the culture of healthcare systems and to achieve excellence in providing care for First Nations peoples. This will be addressed through provision of cultural safety training and creating a community of practice of culturally safe clinicians.

‘Ask the Specialist Plus’ is a structured programme developed by the Communicate Study team to promote antiracism within NT hospitals. It is an 8-week programme based on the ‘Ask the Specialist’ podcast [37]. The ‘Plus’ refers to added activities to support critical reflection on the contents of the podcast. Participants listen to a podcast episode (< 18 min) and then attend a 1-h small-group facilitated discussion with a topic focus (Table 5) during the team’s usual weekly teaching slot. Group size is preferably less than 25 to encourage participation and sharing of ideas in a safe, supportive environment. Facilitators can use materials and slides prepared by V. K. and S. Y. M. as a prompt for discussion points. Embedding ‘Ask the Specialist Plus’ in allocated in-service and clinical teaching times ensures that cultural safety training is valued as much as other clinical skills, and momentum can be amplified as the whole team (students through to directors and multidisciplinary healthcare providers) are all upskilled together, to put learning directly into practice on the wards. This programme was piloted in 2021 in Royal Darwin Hospital’s Department of Women and Children’s Health, and endocrinology departments, demonstrating feasibility and acceptability. Preliminary findings from feedback surveys indicated high satisfaction and a request for more such training (unpublished).

**Table 5:** ‘Ask the Specialist Plus’ training program weekly discussion topics'

Week	Topic	Format
1	Introduction to cultural safety	1-h facilitated discussion Slide presentation developed to support consistency in approach between different facilitators
2	Get to know your patient	Listen to podcast episode in own time and then participate in 1-h facilitated discussion with slide presentation
3	Communicating with your patient	
4	Communicating with interpreters	
5	Patient-centred care	
6	Informed consent	
7	Recognising and addressing racism	
8	Perspectives on health and wellbeing	

**Fig. 3:**
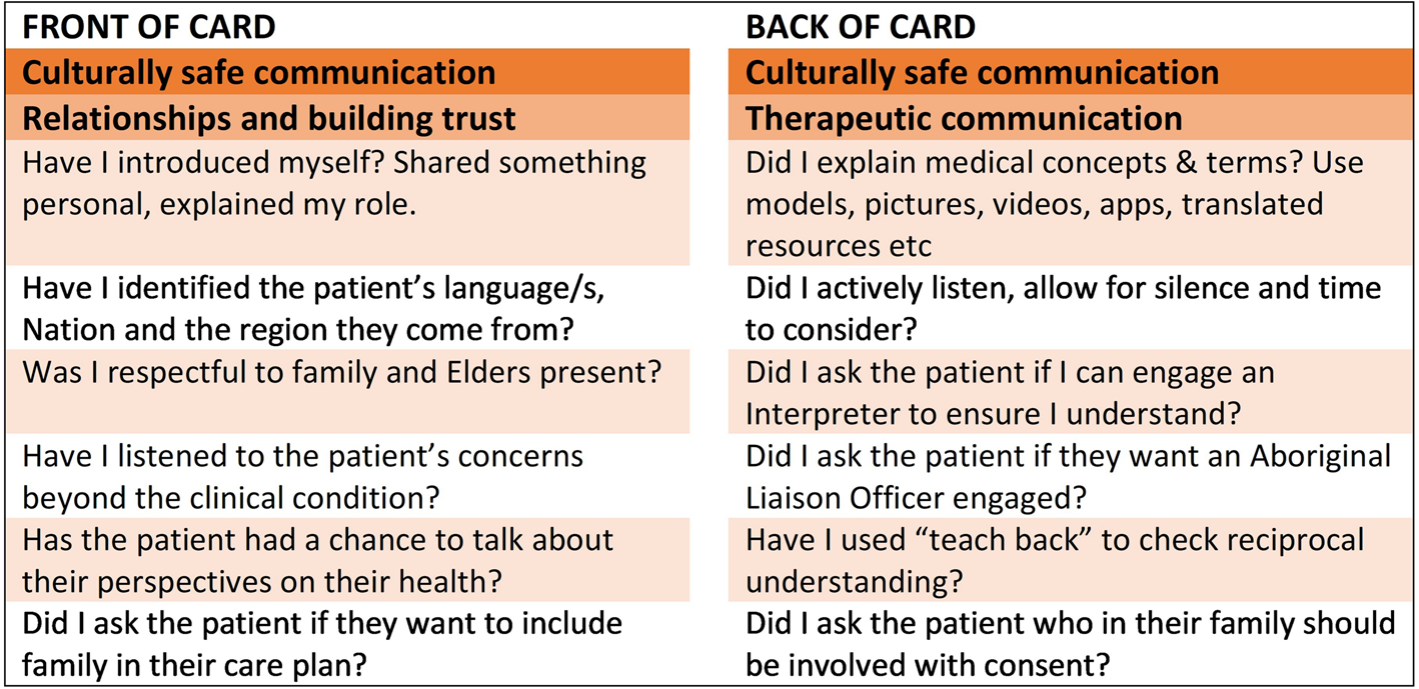
Cultural safety checklist

The staff who attend the training will also receive a printed and laminated cultural safety communication checklist (8 cm by 8 cm square) to be attached to staff identification lanyards (Fig. 3). The checklist was inspired by work undertaken with clinicians in New South Wales, Australia [38], who identified the need to have a self-assessment checklist to guide their practice with First Nations families. We adapted the checklist for use in Top End hospitals, in consultation with the developers, and it was endorsed for piloting by the NT Aboriginal Health Committee.

In addition to ‘Ask the Specialist Plus’, a community of practice of culturally safe clinicians we refer to as champions of cultural safety will be supported by a chat platform and seminars provided by invited experts, detailed below.

### Strengthening the tools and strategies that underpin culturally safe practice

#### Improve the demand for Aboriginal interpreters and Aboriginal health practitioners

A goal of the Community Study Partnership is expansion and effective incorporation of First Nations workforce in health. Demand for this is driven in part by the knowledge and attitudes of mainstream healthcare providers as they gain better understanding of the limitations of biomedical approaches to address First Nations patient needs and of the value of engaging interpreters and Aboriginal health practitioners (Aim 2a). Demand will be promoted by ‘Ask the Specialist Plus’ training in communication and cultural safety and by champions of cultural safety working directly in patient care roles.

‘Champions’ will participate in a chat group hosted on WhatsApp Inc. (WhatsApp Messenger, Meta Platforms) to discuss matters relating to cultural safety and will be invited to seminars approximately 2 monthly. These activities will create a community of practice which values anti-racism approaches in clinical medicine and broader healthcare. Members will comprise multidisciplinary healthcare providers (nurses, doctors, allied health; Indigenous and non-Indigenous). A ‘snowball’ (word of mouth) strategy will be used to expand membership. Members can silence or exit the group as they wish. APR, VK and the research assistant(s) will moderate the group and contribute conversational prompts and responses. The chat platform will provide a forum to share observations and questions about working in the NT intercultural healthcare context and share ideas, articles and other resources.

The project team will also use opportunities to advocate for cultural safety and use of Aboriginal interpreters at teaching sessions, Grand Rounds, clinical handovers, through presentations to hospital committees and preparation and dissemination of policy and practice briefs arising from research findings.

#### Improve the supply of Aboriginal interpreters and Aboriginal health practitioners willing and able to work in the hospital environment

Fulfilling Aim 2b requires the creation of a culturally safe workplace, with mentoring and support for career pathways in health interpreting and healthcare for First Nations peoples. Interpreters can be employed prior to gaining qualifications, using an on-the-job learning model to ultimately gain certification through the National Accreditation Authority for Translators and Interpreters (NAATI). A major new commitment from NT Health for this partnership project is to directly employ interpreters representing some of the commonly spoken languages, to complement the service provided by the external NT Aboriginal Interpreter Service. On-site availability will improve visibility and availability. Creation of a cohesive, supported team will help mitigate the intimidating nature of the work environment [5, 9].

Education in health concepts and hospital processes for interpreters to improve confidence will be coordinated initially by the study team with the plan to hand this role over to hospital education units. Interpreter educational needs are based on problems and questions the interpreters have identified and will be supported by the development of a Plain English Dictionary of health words. As in Aim 1, cultural safety is pivotal — we hypothesise that a more culturally safe workforce achieved through scale up of education and mentoring will improve interpreter experience.

#### Integrate supply and demand through efficiency and effectiveness strategies

Processes for booking an interpreter will be simplified. Lanyards providing contacts for hospital interpreters and the Aboriginal Interpreter Service will be distributed. Working with Interpreter Training will be built into ‘Ask the Specialist Plus’ training sessions for healthcare providers. It covers who needs an interpreter, ‘teach back’ [39] to ensure comprehension, non-judgemental framing of the need for an interpreter and briefing/debriefing of interpreters. Strategies will be explored to optimise workflow to facilitate efficiency in accessing interpreters across hospital departments.

#### Evaluation strategy

The RE-AIM model which will be used for overarching programme evaluation [20, 22] (Table 4) meshes well with the TIDieR checklist (Supplementary Table). RE-AIM evaluation will incorporate analysis of interviews and observations, time-series analysis of hospital admissions, interpreter bookings data and costs and training survey results, with application of the Kirkpatrick model of learning to evaluate training [10, 40]. The Kirkpatrick model describes 4 domains for assessing quality and outcome of education: level 1: reaction (satisfaction, engagement); level 2: learning (knowledge and skills obtained); level 3: behaviour (application of knowledge/skills); and level 4: results (benefit to patients).

#### Quantitative data collection

Data sources will comprise Admitted Patient Care datasets from participating hospitals, Aboriginal Interpreter Service records of interpreter bookings and completions and bookings data from hospital-based interpreters.

#### Admitted patient care datasets

Hospital admissions data will be requested from NT Health Analytics for the 2-year baseline period (July 1, 2020–June 30, 2022; Fig. 1) and in quarterly instalments from the start of the activity period until the end of the study (July 1, 2022–June 30, 2026). Inclusion criteria are admissions of people of all ages identifying as Aboriginal or Aboriginal and Torres Strait Islander. We anticipate approximately 20,000 eligible admissions/year. The following variables will be requested: hospital record number, ward, admission date, discharge date, admission code urgency and type (to identify readmissions within 30 days), discharge destination and type (to identify self-discharges), length of stay, ICD-10-AM codes (International Statistical Classification of Diseases and Related Health Problems, 10th revision, Australian Modification) to gauge complexity of admission and AR-DRGs (Australian Refined Diagnosis Related Groups) for costing data. Discharge type will be categorised as deceased, discharged home, transferred to another facility or took own leave (‘Left Against Medical Advice’ [signed themself out] or ‘Take Own Leave’ [left without notifying anyone]). Data will be examined according to individual hospital and aggregated across all hospitals.

Aboriginal Interpreter Service (AIS) booking records for the baseline and follow-up periods to be collected are as follows: interpreter booking request date, language, ward, name (for linkage purposes) and job fulfilled or not. AIS booking data will be examined to explore patterns of uptake related to wards, hospitals, language/community groups, gender and age.

Bookings for interpreters employed directly through health services (‘Communicating for Safety officers’) will also be accessed, including client hospital record number, date, language and nature of the job (e.g. informed consent; end of life discussion).

#### Quantitative data analysis

The linked dataset of hospital admissions and interpreter bookings will be analysed using interrupted time-series analysis to examine change in gradient and intercept from the baseline to intervention periods in each outcome. Models will be fitted to estimate slopes and differences between slopes, as we have done previously using unlinked data [6]. Analyses using the linked dataset will compare outcomes in First Nations patients who did or did not have access to an interpreter during a given hospitalisation, using logistic regression analysis or mixed-effects models accounting for patient variables.

Economic evaluation will be conducted from the payer perspective and include the linked APC and Aboriginal Interpreter Service datasets and NT Health unit expenditure reports, as well as ICD-10-AM codes and AR-DRGs. ICD-10-AM codes will illustrate complexity, and DRGs will provide the re-imbursement cost for admission (under activity-based funding [41]) and the median and inlier/outlier values for length of stay. These will be used to compute the consequences of self-discharge, for example whether self-discharge occurred early in a complex admission compared to the anticipated length of stay for that diagnosis, therefore likely to have major consequences and high chance of readmission. Additional analysis will compare length of stay to the national average, before and during the study. Decreases in length of stay (not associated with early discharge) are likely to have a costbenefit impact on the health system. However, increased length of stay may ultimately have a cost benefit if associated with decreased readmission rates or improved health outcomes.

Service and salary costs associated with interpreter use and staff time to undertake education and mentoring will be calculated using expenditure reports. Based on the time-series modelling, compiled costs will be input into the models to examine expenditure trends before and during intervention. Probabilistic sensitivity analysis will be used to characterise parameter uncertainty in relation to the level and slope of model outputs (expected hospitalisation rates and costs). Generalised linear modelling will be used to determine variables that have a potential influence on costs (such as hospital, ward, language, age, gender). Incremental cost differences between the projected expenditure trends before and post intervention at years 2 and 4 will be calculated. Incremental costs will demonstrate impact and sustainability of the model.

#### Qualitative data collection

Qualitative analyses will explore impacts of study activities on First Nations patients, interpreters and healthcare providers and their interactions with each other, during project implementation compared with baseline data. Data sources will include interviews, observational field notes and journals. Collection and analysis of qualitative data will be an opportunity for First Nations employees to develop their research skills. A core group of English and Aboriginal language speakers will undertake training to conduct interviews, translation and analysis.

Individuals motivated to improve culturally safe communication in healthcare will be purposefully sampled, in keeping with PAR approaches. Patients, interpreters and healthcare providers who can provide “information rich cases” which exemplify dysfunction and expose systemic issues to be addressed and opportunities and strategies for change will be invited to participate [25, 42]. Logical generalisations relating to systemic issues can be made from a small amount of in-depth evidence. The intention in gathering such data which explores the insider perspectives of key informants is twofold: to evaluate the impact of study activities and to provide informative data back to the partner institutions on strategies for improving quality of care [43].

#### Patients

Cultural safety will be assessed through in-depth interviews with patients in their preferred language. Patients and families will be invited to yarn with researchers about their hospital experience [44]. A yarning guide has been developed to facilitate a semi-structured discussion about what matters to patients regarding communication and cultural safety when seeking healthcare in the NT. These yarns will be conducted by a bilingual researcher, or by an English-speaking researcher with an interpreter as required, and audio recorded with permission. Inclusion criteria are as follows: First Nations Australian, receiving inpatient or outpatient care at a participating hospitals and/or is a trusted family member of a patient and can provide informed consent.

Patients may prefer a group discussion including their accompanying escort or other family members, rather than a one-on-one interview. Therefore, relatives, next of kin or carers will also be eligible to participate if the patient wishes. Guardians of paediatric patients or of patients with disabilities will also be eligible to participate to ascertain their perception of the extent to which the experience for them and the patient was culturally safe. Written, informed consent will be obtained from each participant using a form written in English, with interpreter explanation as required. Patient participants will also be asked for consent to record their hospital record number for the purpose of cross-checking points arising from the interview against medical records should that be required. Issues of concern raised by patients will be escalated appropriately, if they agree, through the treating medical team or the patient advocate.

A purposive sampling approach will be used with maximum sample diversity (patients with diverse first languages including English; who need and did/did not have access to an interpreter; of different genders, age and care type). Potentially eligible participants will be identified through consultation with Interpreters, Aboriginal Liaison Officers and clinicians (including the Cultural Safety Champions) and community contacts. Potentially eligible participants will be approached by a researcher in their first language to discuss the purpose of the study and seek consent.

Approximately, 10 patient participants will be interviewed prior to commencement of ‘Ask the Specialist Plus’ training and approximately 3 per year for the remaining 4 years, providing at least 20 in-depth patient interviews. A subset of patient participants will be invited to participate in follow-up interviews either within a single admission or across admissions. This will include patients with a prolonged stay, or who access healthcare repeatedly for chronic conditions (e.g. dialysis who can allow a rich story to be fully explored, as well as providing the chance to review research findings with the participant and check interpretation [4]). Long-term patients provide greatest sensitivity to change within health systems.

#### Interpreters

Interpreters (± Aboriginal Health Practitioners) will be invited to participate in semi-structured interviews at baseline and during study activity implementation. Inclusion criteria will comprise interpreters or Aboriginal Health Practitioners employed currently or recently or at a participating hospital, who provide written, informed consent. We will interview 5 interpreters at baseline and at one or two follow-up time points, providing approximately 15 interviews. Interviews will explore how interpreters perceive their role, healthcare providers and systems, career pathway, workplace support and perceptions of institutional cultural safety. Based on previous work [5] and ongoing conversations, interpreters play multiple roles in the hospital including as cultural brokers and providing ‘welfare checks’; interviews will provide insights into the breadth of their roles.

#### Healthcare providers

Diverse clinicians (nursing, medical, allied health) from different cultural backgrounds and hospital departments will be invited to participate (approximately 10/year; 50 in total) in audio-recorded interviews/observations eligibility including the following: working currently or within the last 3 months at a participating hospital and providing written, informed consent. Participants will be recruited through the networks of the investigator team and snowballing. The interview guide will focus on perceptions of hospital culture and their own culture in engaging with First Nations patients, barriers and enablers to intercultural communication, power, racism and engagement in reflective practice. Our previous research has found in-depth interviews with an external researcher provide doctors with opportunities to critically reflect on medical and hospital culture without fear of retribution from their employer or judgement from colleagues [9]. Reflective interviews assist healthcare providers to develop critical consciousness, required to instigate change. Interviews will also seek to capture attitudinal and behavioural changes relating to participation in ‘Ask the Specialist Plus’ and feedback on the usefulness of the cultural safety checklist. Healthcare providers will also be encouraged to keep a journal to encourage further reflection and track progress. Suggestions will be sought from healthcare providers about how to address systems issues.

Healthcare providers consenting to interviews in relation to the ‘Ask the Specialist Plus’ training evaluation will be observed by researchers interacting with staff, patients and families. Observations will occur approximately 1 month before and within 2 months after training. This will allow researchers to observe if training participants were able to maintain any attitude and behaviour change hypothesised by trainers and as participants may indicate in their surveys. An observation guide will assist researchers to focus on key features of the interaction relating to culturally safe communication. Researchers will describe in writing the physical environment, note who was present, if First Nations staff were part of the interaction, and will observe and document healthcare providers’ verbal and non-verbal communication style. Observational data helps to guide reflective interviews with healthcare providers, is analysed as data alongside interviews and assists researchers to better understand the systemic issues healthcare providers face.

#### Qualitative data analysis

Supporting the transformative goals of PAR, a critical theory lens which examines power relations embedded in social, political and cultural contexts, will guide analysis [45]. Interviews conducted in English will be transcribed verbatim. Interviews conducted in a First Nations language will be translated into English by a native speaker/researcher using meaning-based interpreting [46]. Key words or concepts expressed in First Nations languages will be retained to ensure cultural meaning is not lost in translation. During analysis, First Nations researchers are cultural brokers drawing on their knowledge to ensure stories are accurately represented and understood. Data from patients, interpreters and healthcare providers will be uploaded to NVivo software (QSR International Pty Ltd., 2020) and inductive narrative analysis undertaken to identify key turning points in people’s stories (epiphanies) or triggers leading to behaviour change [47]. Data from surveys pertaining to training (interpreter health education and ‘Ask the Specialist Plus’) will be deductively organised into categories identified in Kirkpatrick’s training evaluation framework: reaction, learning, individual behaviour change and organisational impact [40]. After the data have been analysed and reconstructed into draft stories, participants will be contacted to review how their story has been told. This ensures stories are verified and gives participants a final opportunity to add details.

In PAR projects, participants sometimes wish to use their real name. We will ask participants to choose whether and how they would like to be identified in the research. Pseudonyms can protect participant identity, but they can also erase the connection between an individual and their knowledge. In our previous research, some participants used their names to ensure knowledge shared was attributable to them, thereby maintaining sovereignty over ideas [9].

#### Activity and implementation logs

A log of project activities such as milestones in approval processes (ethics, research governance, inter-institution agreements), presentations, data collection, training sessions and other key events will be recorded to help inform reporting to the funders and ethics committee, the study newsletter, and provide data for the evaluation (e.g. ‘implementation’; see Table 4). The way in which intervention components may be tailored or modified during implementation will be documented in a study implementation log. A regular electronic study newsletter will be distributed using Mailchimp™ Application Programming Interface. Proportion of recipients who open the email will be rerecorded.

#### Staff surveys

Healthcare providers will be invited to participate in paper-based or online surveys after participating in ‘Ask the Specialist Plus’ [21] training. This will determine responses across the 4 elements of the Kirkpatrick model of learning [10, 40].

## Discussion

The goal of this study, to achieve excellence in culturally safe care for First Nations peoples to improve health outcomes, is widely endorsed, yet there are significant challenges to accomplishing it. The Communicate Study Partnership addresses these challenges in the northern Australian tertiary care context through pragmatic solutions. Preliminary data suggest that our approaches have high likelihood of achieving the desired outcomes [4, 6, 9, 15]. However, changes in health system culture and healthcare provider behaviour to achieve better cultural safety and patient experience can only be sustained if these new approaches become embedded as ‘the new normal’, in a way that is robust to staff turnover, which is very high in the NT [48]. Project design therefore focuses on sustainability and maintenance of activities beyond the life of the project through effective partnership with service delivery organisations.

Progress is underway in each activity domain, and baseline data collection has occurred. Royal Darwin Hospital is employing Aboriginal interpreters and a coordinator who serves as a mentor and support person. The hospital-employed interpreters currently cover six Aboriginal languages: Yolŋu Matha languages, Kunwinjku, Kriol, Maung, Murrinh-Patha and Murrinh-Kura. Other language interpreters can be accessed using a bookings process through the NT Aboriginal Interpreter Service.

‘Ask the Specialist Plus’ sessions are being delivered at Royal Darwin Hospital in 2023, during which time new facilitators will be upskilled, and facilitators for the sites outside Darwin will be sought. There are currently 87 cultural afety champions on the chat platform, on which messages are posted most days.

This project has the potential to substantially improve First Nations patients’ experience of care and health outcomes. Culturally unsafe care has broad adverse outcomes, while good communication is associated with better outcomes [3, 6, 49]. Healthcare systems need to identify ways in which they contribute to disparities in health outcomes experienced by culturally diverse populations. Failure to provide care in a patient’s first language is a clear example. Our project responds to this with effective, innovative and durable approaches to transform the culture of healthcare systems to achieve excellence in culturally safe care for First Nations people. Accompanying benefits will include strengthening the First Nations workforce and strengthening healthcare providers’ capacity to deliver culturally competent care which will contribute to reducing the stressors associated with ineffective communication. Healthcare providers experience burnout and high turnover when engagement with patients is poor [50]; measures which addresses this are likely to foster greater healthcare provider retention, a major need in northern Australian healthcare.

## Conclusion

Project activities are locally targeted and context-specific, but our project approach and findings also have transferability nationally and internationally. If healthcare institutions and the individuals working in them can transform the provision of care in innovative ways that depart from traditional medical structures, to create genuine cultural safety, this would provide a powerful mechanism in closing the gap in health outcomes experienced by First Nations Australians.

### Supplementary Information

**Additional file 1:** **Table.** Description of study intervention activities according to the Template for Intervention Description and Replication (TIDieR) checklist.

## Data Availability

Not applicable.

## Abbreviations

AIS: Aboriginal Interpreter Service
CQI: Continuous quality improvement
GDH: Gove District Hospital
HREC: Human research ethics committee
KH: Katherine Hospital
RE-AIM: Reach, Effectiveness, Adoption, Implementation and Maintenance
RDH: Royal Darwin Hospital
TIDieR: Template for Intervention Description and Replication
NAATI: National Accreditation Authority for Translators and Interpreters
NT: Northern Territory
NSQHS: National Safety and Quality Health Service
PAR: Participatory action research
